# Interrelationships between sarcopenia, bone turnover markers and low bone mineral density in patients on hemodialysis

**DOI:** 10.1080/0886022X.2023.2200846

**Published:** 2023-04-25

**Authors:** Yilin Wang, Wenxia Ma, Jianhong Pu, Fengling Chen

**Affiliations:** a The Blood Purification Center, The First Affiliated Hospital of Soochow University, Suzhou, P. R. China; b Quality Management Dept, The First Affiliated Hospital of Soochow University, Suzhou, P. R. China; c The Center of Health Management, The First Affiliated Hospital of Soochow University, Suzhou, P. R. China

**Keywords:** Bone mineral density, skeletal muscle mass index, hemodialysis, sarcopenia, osteoporosis, osteosarcopenia

## Abstract

**Background:**

Hemodialysis (HD) patients are at risk for sarcopenia (SP) and bone loss, which may impact falls and bone fragility and lead to poor prognosis. Patients with HD and those with osteoporosis (OP) are still underdiagnosed and untreated. The aims of the present study were to evaluate the factors that affect bone mineral density (BMD) loss in HD patients, and explore traditional and novel approaches to manage chronic kidney disease–mineral-bone disorder (CKD-MBD).

**Methods:**

Patients who underwent regular HD at the First Affiliated Hospital of Soochow University were retrospectively evaluated. According to the WHO osteoporosis criteria, patients were categorized into three groups: normal BMD, osteopenia, and osteoporosis. Demographic and clinical data, skeletal muscle mass, and bone turnover markers(BTM) were compared between the three groups. The correlation between bone density and muscle mass was calculated, and related risk factors were analyzed.

**Results:**

This study enrolled 130 HD patients, 36 patients were diagnosed with sarcopenia (27.7%), 44 patients were diagnosed with osteopenia (33.8%), 19 patients were diagnosed with osteoporosis (14.6%), and 23 patients were diagnosed with osteosarcopenia (17.7%). The SMI was positively correlated with the BMD of the lumbar spine (*r* = 0.23, *p* < 0.01) and femoral neck (*r* = 0.22, *p* < 0.05). In ordinal logistic regression analysis, the odds ratio (OR) for low BMD was high for patients with sarcopenia (OR = 5.894, 95% CI 1.592–21.830, *p* < 0.01), older age (OR = 1.095, 95% CI 1.041–1.153, *p* < 0.001), higher TRACP-5b levels (OR = 1.597, 95% CI 1.230–2.072, *p* < 0.01), and lower 25-OH vitamin D levels (OR = 0.631, 95% CI 0.544–0.733, *p* < 0.001).

**Conclusion:**

The preservation of skeletal muscle mass could be important to prevent a BMD decrease in HD patients. Adequate intake of vitamin D and control of TRACP-5b levels will help reduce the occurrence and progression of osteopenia/sarcopenia in HD patients.

## Introduction

Chronic kidney disease–mineral and bone disorder (CKD-MBD) are common in hemodialysis patients, with not only disturbances of calcium, phosphate, parathyroid hormone (PTH) and vitamin D metabolism but also cardiovascular calcification and bone abnormalities, leading to an increased incidence of fracture and other poor outcomes [1]. CKD stage 3a-5D patients have low bone mineral density and reduced mechanical strength, resulting in a significantly higher fracture risk (1.5–2 times higher than that of the general population) [2]. In addition, fractures tend to occur at an earlier age in them [3], with increased associated morbidity and mortality (3.7 times higher in dialysis patients than in the overall DOPPS population) [4]. When renal function falls in dialysis patients, phosphorus levels increase, resulting in elevated PTH. Nephrologists often prevent bone loss by lowering the patient’s serum phosphorus and PTH levels. Even if these efforts are made, dialysis patients are more likely than the general population to have osteoporosis [5,6]. These patients need additional interventions beyond traditional management.

Meanwhile, as part of HD, patients often suffer from sarcopenia, a loss of strength and mass in their skeletal muscles [7]. Osteoporosis and sarcopenia are both associated with poor long-term survival [7,8]. Binkley and Buehring [9,10] proposed a syndrome called osteosarcopenia, which is characterized by the coexistence of osteopenia and osteoporosis. But the diagnostic criteria for this syndrome are unclear, which contributes to its poor understanding [10]. It is crucial to understand its pathophysiology and diagnosis, as well as its management. However, the majority of studies examining the association between muscles/strength and bone have been conducted in the general population, rarely in HD patients. The aims of the present study were to clarify the incidence of sarcopenia and bone loss in HD patients, evaluate the factors that affect BMD loss, and explore traditional and novel approaches to manage CKD-MBD. Accurate assessment and timely detection of sarcopenia and bone loss in HD patients is crucial for providing quick and adequate multidisciplinary therapy to improve survival.

## Methods

### Study population

330 patients who underwent regular hemodialysis were screened for this study in the First Affiliated Hospital of Soochow University from January 2022 to October 2022. Adult patients were eligible to participate in the study if they: (1) aged 18–90 years; (2) underwent regular maintenance hemodialysis for >3 month, 3 times a week with 4 h sessions; and (3) were able to complete the interview. Exclusion criteria: (1) acute infection; (2) history of parathyroidectomy; (3) other major diseases (such as malignancy); (4) taking steroids, anti-resorptive drugs (bisphosphonates), contraceptives, or calcitonin; (5) were bedridden or unable to undergo the examination; (6) declined to participate in this study. Healthy control (*n* = 75) samples were selected from the Center of Health Management in our hospital. This study was approved by the ethics committees of The First Affiliated Hospital of Soochow University (2022-160), and this study was performed in accordance with the ethical standards as laid down in the 1964 Declaration of Helsinki and its later amendments or comparable ethical standards. All participant’s data were kept confidential.

Among 330 HD patients from HD centers for this study (January 2022 to October 2022), a total of 130 subjects were finally recruited for analyses in the present study.

### Data collection

Demographic and clinical data, including age, sex, body mass index (BMI, kg/m^2^), comorbidities, etiology of kidney disease, dialysis vintage (months) and dialysis modality were collected. The presence of diabetes mellitus (DM), and vitamin D, calcium supplement, and calcimimetic use were noted.

Peripheral blood samples were obtained in a fasting state in both hemodialysis patients and healthy control participants, within a week of DXA. Blood was taken through the arteriovenous fistula or graft before HD, except plasma brain natriuretic peptide (BNP), which was collected from patients at the end of HD. Routine laboratory parameters for CKD patients (hemoglobin, total cholesterol, triglyceride, phosphorus, calcium, total alkaline phosphatase (ALP), albumin, intact PTH (PTH), 25(OH) vitamin D (25(OH)D) and β 2 microglobulin (β 2-MG) levels were analyzed at the University Hospital Central Laboratory. Serum bone alkaline phosphatase (bAP) and tartrate-resistant acid phosphatase 5b (TRACP-5b) samples were taken before the dialysis session, kept at −20 °C, and sent to a clinical laboratory of KingMed Center for Clinical Laboratory Co., Ltd for the assays, bAP was using a chemiluminescent immunoassay, and TRACP-5b was using enzyme-linked immuno sorbent assay. Venous blood samples for BNP analysis were using i-STAT BNP POC device (Abbott, East Windsor, NJ).

### Assessment methods

#### Sarcopenia

Body composition characteristics which include fat-free mass (FFM), soft lean mass (SLM), and skeletal muscle mass (SMM) values were measured by multifrequency segmental bio-impedance (MFBIA) (InBody 710 instrument, BioSpace, Seoul, Korea), which was regularly serviced and calibrated. We performed BIA in healthy controls and HD patients while they were fasting and after they urinated to determine normal hydration status, using a standardized protocol [11].

HD patients were measured 30 min after dialysis treatment to allow for re-equilibration on a midweek session [12,13]. Based on published data [14], subjects were required to reach dry weight(DW) with a post-dialysis ECW/TBW (Extracellular water/total body water) ≤0.40 (suggested by the manufacturer, Biospace, Seoul, South Korea). Appendicular skeletal muscle mass (ASM) was calculated as the sum of the muscle mass of the arms and legs.ASM was divided by height in meters squared as a standard adjustment approach: ASM index (ASMI, kg/m^2^).

Handgrip strength was measured using a myometer (EH101; Camry, Zhongshan, China). Two separate trials were performed using the non-fistula arm of the subject with a rest period of at least 1 min between trials, and the maximum values were recorded. Healthy participants were measured using the same equipment and in the same manner as HD patients.

The usual walking speed was measured as an index of physical performance. The time taken (s) to walk 6 m at normal walking speed was recorded, and the usual gait speed was calculated.

Sarcopenia was defined as proposed by the Asian Working Group for Sarcopenia (AWGS) [15], with cutoff values for muscle mass measurements (7.0 kg/m^2^ for men and 5.7 kg/m^2^ for women by using bioimpedance analysis), handgrip strength (<28 kg for men and <18 kg for women), and usual gait speed (<1 m/s). Sarcopenia was diagnosed when a patient had a reduced SMI combined with lower hand grip strength and/or slower speed.

#### Osteoporosis

Lumbar spine and femoral neck bone mineral density (BMD) values were determined using dual-energy X-ray absorptiometry (DXA; GE-Lunar). The measurements were performed after dialysis, according to the manufacturer’s recommended standard analysis procedures for the lumbar spine (vertebrae L2–L4) and hip femoral neck. In the same way as HD patients, healthy controls were also measured. Instrument quality control was performed regularly. Bone mass density, expressed in gm/cm^2,^ was measured at the lumbar spine, total hip, femoral neck.

According to WHO criteria, patients were categorized into three groups: normal bone mineral density with a *T*-score no less than −1.0 SD, osteopenia with a *T*-score between −1.0 SD and −2.5 SD, and osteoporosis for patients with a *T*-score less than −2.5 SD in at least one of these sites: lumbar spine, femoral neck or total hip [16].

### Definition of osteosarcopenia

Osteosarcopenia was defined as (1) low bone mineral density (BMD) [T score less than −1 standard deviation (SD)] combined with sarcopenia and (2) osteoporosis (BMD T score of −2.5 SD or less) combined with severe sarcopenia. Thus, osteosarcopenia is a syndrome defined by the combination of low bone density (osteopenia/osteoporosis) and sarcopenia [10,17].

### Statistical analysis

We selected the study conducted by Montenegro et al. [18] evaluating osteosarcopenia in patients with non-dialysis dependent chronic kidney disease aiming to assess the risk factors of low BMD. These authors observed a 17.5% sarcopenia prevalence of low BMD (10/57), and 2.4% sarcopenia prevalence of low BMD (2/83), sampling analysis considered type I error alpha = 0.05 and type II error beta = 0.10, calculated from the sample size of the case-control studies, resulting in a minimum sample size of 81 patients. Given the possibility of data being missing, we included a larger sample size.

Mean ± standard deviation expressed continuous variables when normal distribution and the median (inter-quartile range) had expressed them when the distribution was not normal. Qualitative variables were expressed as frequencies. Comparisons of continuous variables were performed according to distribution pattern by ANOVA or Kruskal–Wallis tests and by independent samples T-tests or Mann–Whitney tests. Differences in proportions were compared using the chi-square test. Pearson’s or Spearman’s correlation coefficients (according to continuous variable distribution patterns) were calculated to analyze the degree of association between two variables. Ordinal logistic regression analysis was performed to explore possible risk factors (as independent categorical variables) associated with BMD status (normal BMD, Osteopenia, Osteoporosis) as a categorical dependent variable. The results are presented as odds ratios (ORs) with 95% confidence intervals (CIs). Unadjusted and adjusted analysis Statistical significance was considered when *p* < 0.05. All analyses were performed using the SPSS 26 package.

## Results

### Baseline characteristics of all patients on maintenance dialysis

**Figure 1.**
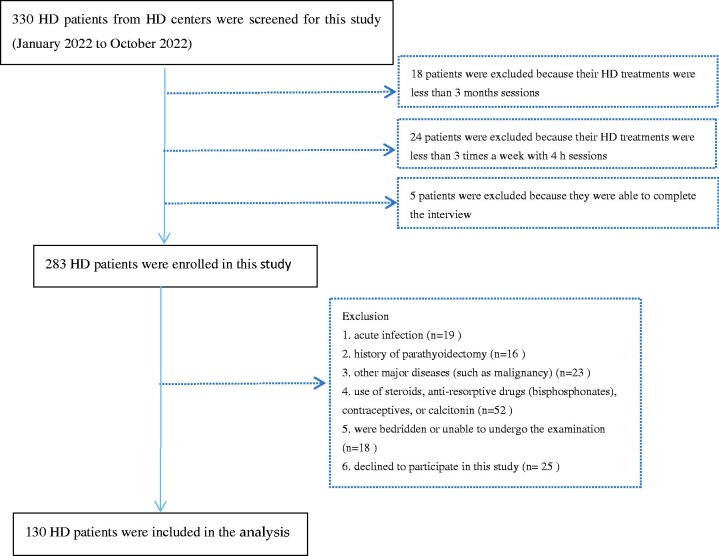
Study flow, including patient enrollment and outcomes.

From January 2022 to October 2022, a total of 330 patients who underwent regular hemodialysis were screened, and 130 of them had sufficient data in the final analysis (Figure 1). Additionally, there were 75 healthy controls in the control group. The study comprised 205 subjects, of whom 125 were categorized into the 18 to 59 years age group and 80 into the 60 years old and above (60+) age group (see Table 1). Comparisons were made by age groups, no significant differences were observed in age, male proportion, the ratio of ECW/TCW, bAP and the incidence of sarcopenia between the hemodialysis group and control group (*p* > 0.05).

**Table 1.** Characteristics of participants between hemodialysis patients and control according to age subgroup.

	Age 18-59 (*n* = 125)			Age 60<(*n* = 80)		
	Case group (*n* = 83)	Control group (*n* = 42)	*P*	Case group (*n* = 47)	Control group (*n* = 33)	*P*
Male (*n* ,%)	47 (56.6 %)	25 (61.0 %)	0.644	25 (53.2 %)	16 (48.5 %)	0.678
Age (years)	46.44 ± 9.80	48.95 ± 7.93	0.152	67.36 ± 4.83	67.91 ± 4.03	0.595
BMD subgroup (*n*,%)						
BMD Normal	52 (62.7 %)	37 (88.1 %)	0.009	15 (31.9 %)	23 (69.7 %)	0.003
Osteopenia	25 (30.1 %)	3 (7.1 %)		19 (40.4 %)	7 (21.2 %)	
Osteoporosis	6 (7.2 %)	2 (4.8 %)		13 (27.7 %)	3 (9.1 %)	
bAP (ng/mL)	17.46 ± 10.62	13.59 ± 4.33	0.115	16.76 ± 9.71	13.04 ± 4.61	0.156
TRACP-5b (mU/dL)	3.42 ± 2.01	0.95 ± 0.31	<0.001	3.47 ± 2.09	0.68 ± 0.25	<0.001
ECW/TCWratio	0.375 ± 0.021	0.379 ± 0.007	0.211	0.383 ± 0.028	0.390 ± .007	0.229
Sarcopenia (*n*,%)	21 (25.3 %)	4 (9.5%)	0.057	15 (31.9%)	4 (12.1%)	0.061

bAP: bone-specific alkaline phosphatase; TRACP-5b: tartrate-resistant acid phosphatase 5b: ECW/TBW: Extracellular water/total body water.

The distribution frequency of BMD subgroups in patients with hemodialysis was significantly different compared with the control group regardless of the age subgroup (*p* < 0.01). In our study, HD patients in the 18–59 years stratum had a 4.2-fold higher proportion of Osteopenia than control groups (30.1% vs 7.1%) and a 1.5-fold higher proportion of Osteoporosis than control groups (7.2% vs 4.8%). In the 60+ age group, HD patients had a 1.9-fold higher proportion of Osteopenia than control groups (40.4% vs 21.2%) and had a 3-fold higher proportion of Osteoporosis than control groups (27.2% vs 9.1%). Patients on hemodialysis are at increased risk of osteopenia/osteoporosis than the healthy controls. Meanwhile, HD patients had higher TRACP-5b in bone turnover markers than control groups regardless of the age subgroup(*p* < 0.001) (Table 1).

The baseline characteristics of the HD patient are summarized in Table 2. The median age of this population was 26 − 79 (54.12 ± 13.09) years, and 72 (55.4%) of the patients were males. In total, the mean HD duration was 62 (IQR 29.75,118) months, and the mean body mass index (BMI) was 22.4 ± 3.6 kg/m^2^. The main underlying diseases were glomerulonephritis (71, 54.6%), diabetes mellitus (22, 16.9%), hypertension (18, 13.8%)), other diseases including vascular nephropathy (2, 1.5%) and polycystic kidney disease (8, 6.2%), tubulointerstitial nephropathy (4, 3.1%) and unknown etiologies (5, 3.8%).

**Table 2.** Baseline characteristics of all patients on maintenance dialysis.

Characteristics	Overall cohort
Age, mean ± SD years	54.1 ± 13.1
Sex	
Male (n, %)	72 (55.4)
Female (n, %)	56 (44.6)
Height, mean ± SD cm	165.5 ± 8.1
Weight, mean ± SD kg	61.6 ± 11.5
Body mass index, mean ± SD kg/m2	22.4 ± 3.6
Duration of dialysis, months	62 (29.75, 118)
Cause of ESKD (%)	
Glomerulonephritis (n, %)	71 (54.6)
Diabetic nephropathy (n, %)	22 (16.9)
Hypertension (n, %)	18 (13.8)
Vascular nephropathy (n, %)	2 (1.5)
Polycystic kidney disease (n, %)	8 (6.2)
Tubulointerstitial nephropathy (n, %)	4 (3.1)
Unknown	5 (3.8)

ESKD: end-stage kidney disease.

### Comparison analysis among groups according to BMD status

The baseline characteristics of patients on hemodialysis by BMD are described in Table 2. There were 67 patients in the Normal BMD group, 44 patients in the osteopenia group, 19 patients in the osteoporosis group. The sex distribution was similar among groups according to BMD status. There were no differences in the duration of dialysis, serum calcium, serum phosphatase, PTH, β2-MG, ALP or bAP levels (Table 2). Compared with patients with normal BMD, those with osteopenia and osteoporosis were more likely to be older (*p* < 0.001) and have a higher incidence of DM (*p* < 0.001), while they showed higher TRACP-5b and lower 25 hydroxyvitamin D in bone turnover markers (*p* < 0.05) (Table 3).

**Table 3.** Comparison of characteristics classified by BMD status.

	Normal BMD (*N* = 67, 51.5%)	Osteopenia (*N* = 44, 33.8%)	Osteoporosis (*N* = 19, 14.6%)	*P*
Female sex (%)	26 (38.8)	20 (45.5)	12 (63.2)	0.168
Age (years)	48.34 ± 12.91	58.36 ± 8.56	63.89 ± 13.08	<0.001
Duration of dialysis (months)	65(35, 121)	57(20, 82)	70(24, 132)	0.411
Diabetes (*n*,%)	3, 4.5%	12, 27%	7, 36.8%	<0.001
Calcium (mmol/l)	2.34 ± 0.17	2.3 ± 0.22	2.31 ± 0.29	0.248
	2.05 ± 0.6	1.86 ± 0.61	1.82 ± 0.61	
Phosphate (mmol/l)	2(1.64, 2.31)	1.74(1.43, 2.22)	1.82(1.39, 1.96)	0.184
Intact PTH (pg/mL)	308.15(203. 4, 577.8)	248.80(125.54, 447.34)	240(164.1, 384.7)	0.212
ALP (ng/mL)	83.4(64.1, 120)	79.05(68.90, 109.08)	91.9(69.8, 129.6)	0.886
β2-MG (mg/L)	30.87 ± 8.01	29.7 ± 8.62	32.73 ± 6.3	0.382
25(OH)D (ng/ml)	23(18, 27)	13.25(10.70, 17.60)	8.1(5.6, 11)	<0.001
bAP (ng/mL)	13.75(9.18, 20.41)	13.77(10.46, 20.37)	17.65(12.86, 30.57)	0.111
TRACP-5b (mU/dL)	3.17 ± 1.73	3.4 ± 1.73	4.46 ± 3.19	0.049
Fe (μmol/L)	13.46(10.33, 18.47)	12.07(9.07, 15.03)	12.46(8.38, 18.68)	0.109
Ferritin (ng/ml)	154.1(84.6, 350.5)	217.55(130.85, 391.63)	337.2(175.5, 511.3)	0.105
TP (g/L)	71.03 ± 5.95	69.36 ± 4.64	70.61 ± 4.14	0.266
Albumin (g/L)	42.3(40.6, 43.9)	41.20(38.55, 43.33)	41.1(38.8, 43.7)	0.036
Hb (g/L)	121(108, 131)	117.00(111.00, 123.75)	111(105, 121)	0.117
Total cholesterol (mg/dL)	4.11 ± 1.21	4.03 ± 0.93	4.09 ± 0.95	0.925
Triglyceride (mg/dL)	1.65(1.13, 2.56)	1.61(1.15, 1.96)	1.35(0.81, 2.76)	0.646
Prealbumin (g/L)	352.74 ± 86.3	337.88 ± 60.76	325.97 ± 75.36	0.339
WBC (10^9/L)	6.48 ± 2.02	6.64 ± 1.8	6.22 ± 2.6	0.746
hsCRP (mg/L)	4.02(1.47, 8.23)	3.28(1.20, 7.02)	2.41(0.93, 5.53)	0.342
KT/V	1.42 ± 0.28	1.45 ± 0.22	1.55 ± 0.21	0.167
Medication use, *n* (%)				
P binder, *n* (%)				
Ca containing	3 (4.5%)	3 (6.8%)	2 (10.5%)	0.610
Non-Ca containing	6 (89.6%)	38 (86.4%)	17 (89.5%)	0.866
VDRA, *n* (%)				
Alfacalcidol/Calcitriol	37 (55.2%)	24 (54.5%)	10 (52.6%)	0.980
Paricalcitol	12 (17.9%)	3 (6.8%)	2 (10.5%)	0.223
CaSR agonist, n (%)	29 (43.3%)	13 (29.5%)	6 (31.6%)	0.297

BMD: bone mineral density; Ca: calcium; P: phosphate; iPTH: parathyroid hormone; ALP: alkaline phosphatase; β2-MG: β2-microglobulin; bAP: bone-specific alkaline phosphatase; TRACP-5b: tartrate-resistant acid phosphatase 5b; 25(OH)D: 25-hydroxyvitamin D;TP: total protein; Hb, hemoglobin; hsCRP: high-sensitivity C-reactive protein; WBC: white blood cell; Kt/V: single-pool Kt/V; VDRA: Vitamin D receptor activators.

Normal range of PTH: 12–88pg/ml; normal range of β2-MG: 0.9 mg/*L*–2.7 mg/L; normal range of TRACP-5b: 0.5–4.82U/L.

There were no differences in routine laboratory variables, including Fe, ferritin, total protein, hemoglobin, total cholesterol, triglyceride, prealbumin, WBC, hsCRP and KT/V(*p* > 0.05), except albumin (*p* < 0.05). Patients in the osteopenia and osteoporosis groups presented with lower albumin levels than patients in the normal BMD group (*p* < 0.05) (Table 3).

The percentage of patients receiving calcium-containing or non-calcium-containing phosphate binders, the percentage of patients receiving VDRAs and the percentage of patients receiving CaSR agonists were no differences among the three categories in Table 3.

In terms of markers of volume overload, brain natriuretic peptide (BNP) and the ratio of ECW/TBW were similar between the three BMD subgroups (*p* > 0.05), suggesting similar hydration status. The normal BMD group presented with higher height and weight than the osteopenia and osteoporosis groups (*p* < 0.01). However, BMI was similar in each BMD subgroup (*p* > 0.05). Patients in the osteopenia and osteoporosis groups presented with lower fat-free mass (FFM), soft lean mass (SLM), skeletal muscle mass (SMM), appendicular skeletal muscle mass (ASM), ASMI and grip strength values than patients in the normal BMD group (*p* < 0.05) (Table 4).

**Table 4.** Body composition characteristics according to BMD status.

	Normal BMD (*N* = 67, 51.5%)	Osteopenia (*N* = 44, 33.8%)	Osteoporosis (*N* = 19, 14.6%)	*P*
Height (cm)	170(162, 172)	165.50(160.00, 170.00)	158(155, 165)	0.002
Weight (kg)	63.98 ± 12.18	61.26 ± 9.92	53.73 ± 8.81	0.002
BMI (kg/m^2^)	22.78 ± 3.68	22.48 ± 3.45	21.01 ± 3.62	0.171
Body fat mass (kg)	14.9(11.5, 21.1)	15.20(11.55, 20.48)	11(9.1, 15)	0.103
Fat free mass (kg)	47.94 ± 9.75	44.99 ± 7.38	39.95 ± 5.49	0.001
Soft lean mass (kg)	45.26 ± 9.27	42.4 ± 6.86	37.43 ± 5.6	0.001
Skeletal muscle mass (kg)	26.49 ± 5.79	24.75 ± 4.72	21.18 ± 3.35	<0.001
ASM (kg)	20.03 ± 5.02	18.51 ± 4.38	16.03 ± 3.65	0.004
ASMI (kg/m^2^)	7.24(6.34, 7.93)	6.81(6.21, 7.27)	6.21(5.62, 6.77)	0.025
Grip strength (kg)	25.7 ± 6.13	23.4 ± 4.4	17.9 ± 2.3	<0.001
ECW/TBW ratio	0.378 ± 0.011	0.375 ± 0.037	0.385 ± 0.021	0.198
BNP (pg/mL)	366.18 ± 45.63	363.45 ± 67.96	381.68 ± 34.84	0.440

BMI: body mass index; ASM: appendicular skeletal muscle mass; ASMI: appendicular skeletal muscle mass index.

## Associations and correlations between BMD and muscle mass parameters in hemodialysis patients

### Epidemiological and clinical characteristics of the patients

**Figure 2.**
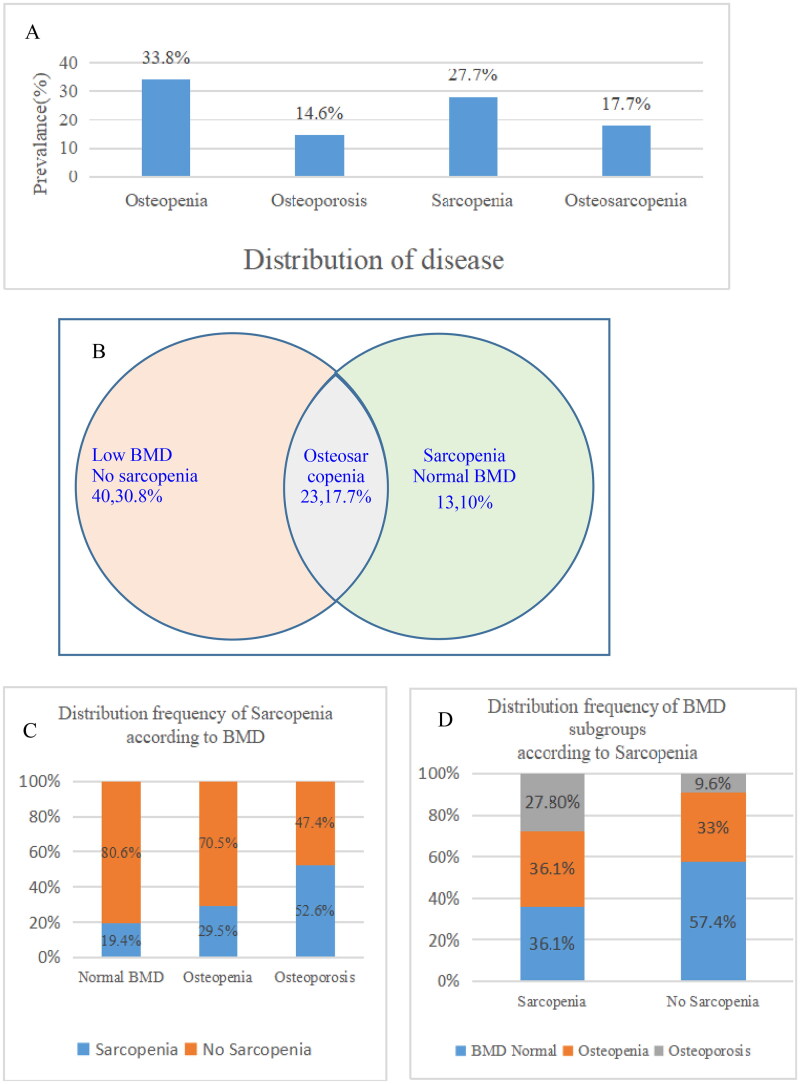
**(A)** Frequency of osteosarcopenia criteria in hemodialysis patients; **(B)** Intersections between low BMD,and sarcopenia. Presented as n (%); **(C)** Distribution frequency of BMD subgroups according to sarcopenia. Chi-square test for comparisons between 2 groups (normal, sarcopenia): *p* = 0.016; **(D)** Distribution frequency of Sarcopenia according to BMD status. Chi-square test for comparisons among 3 groups (normal, osteopenia, osteoporosis): *p* = 0.027.

Among the 130 hemodialysis patients, 36 patients were diagnosed with sarcopenia (27.7%), 44 patients were diagnosed with osteopenia (33.8%), 19 patients were diagnosed with osteoporosis (14.6%). Over 50% of Patients (10, 52.6%) with OP had SP, and the majority of patients (63.9%) with SP had low BMD in Table 4. Osteosarcopenia is defined as the combination of low bone density (osteopenia/osteoporosis) and sarcopenia, the degree of co-existence of SP and low BMD was clarified in our study, 23 patients were diagnosed with osteosarcopenia (17.7%) (Figure 2(A, B)).

The distribution frequency of BMD subgroups in patients with sarcopenia was significantly different compared with the normal group(*p* = 0.016). The distribution frequency of Sarcopenia among the three BMD subgroups (normal BMD, Osteopenia, Osteoporosis) was significantly different(*p* = 0.027) (Table 5, Figure 2(C and D)).

**Table 5.** Intersections between low BMD, and sarcopenia. Presented as n (%).

	Normal BMD (*N* = 67, 51.5%)	Osteopenia (*N* = 44, 33.8%)	Osteoporosis (*N* = 19, 14.6%)	*P*
Sarcopenia(n,%)				
(+)*n* = 36, 27.7%	13 (36.10%)	13 (36.10%)	10 (27.80%)	0.027
(-)*n* = 94, 72.3%	54 (57.4%)	31 (33.0%)	9 (9.6%)	
	Sarcopenia(−) *n* = 94, 72.3%		Sarcopenia(+) *n* = 36, 27.7%	*P*
BMD subgroup(n,%)				
BMD Normal	54 (57.40%)		13 (36.10%)	0.016
Osteopenia	31 (33.00%)		13 (36.10%)	
Osteoporosis	9 (9.60%)		10 (27.80%)	

As presented in Figure 2(C), the osteopenia and osteoporosis groups accounted for a higher proportion of the sarcopenia group than the other group. As presented in Figure 2(D), with decreasing BMD, there was an increased proportion of sarcopenia in the subgroups.

**Figure 3.**
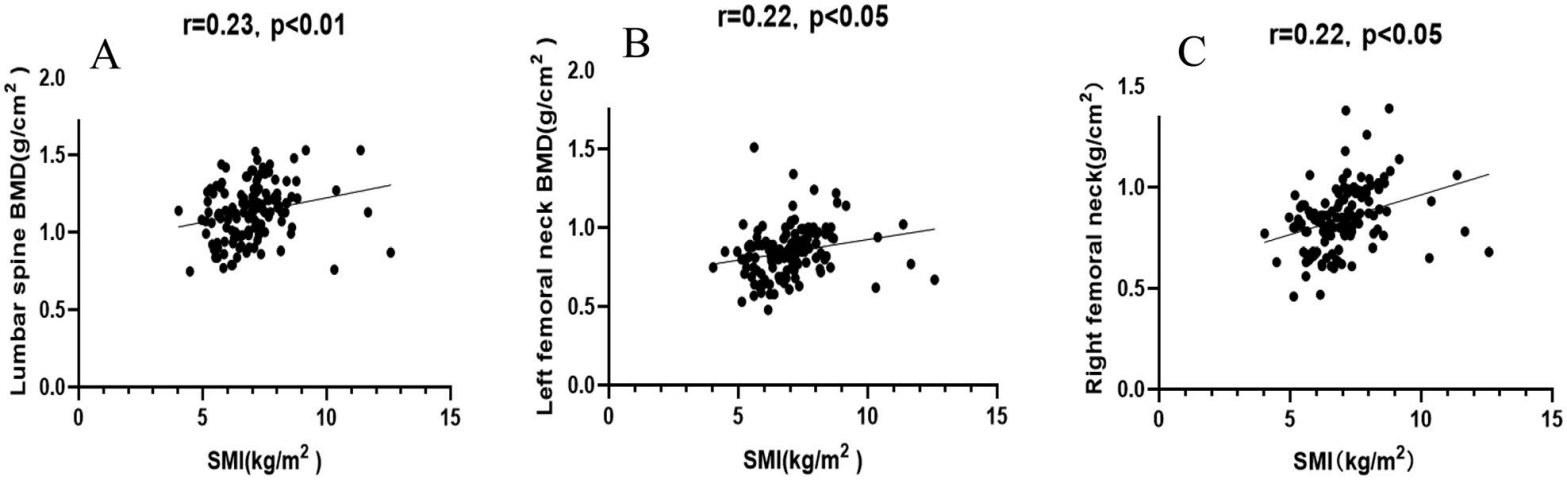
**(A)** Correlation between the SMI and the BMD of the lumbar spine. **(B)** Correlation between the SMI and the BMD of the left femoral neck. **(C)** Correlation between the SMI and the BMD of the right femoral neck.

The SMI was significantly positively correlated with the BMD of the lumbar spine (*r* = 0.23, *p* < 0.01), left femoral neck (*r* = 0.22, *p* < 0.05), and right femoral neck (*r* = 0.22, *p* < 0.05). Low SMI was associated with a higher risk for low BMD in both the lumbar spine and femoral neck (Figure 3).

**Figure 4.**
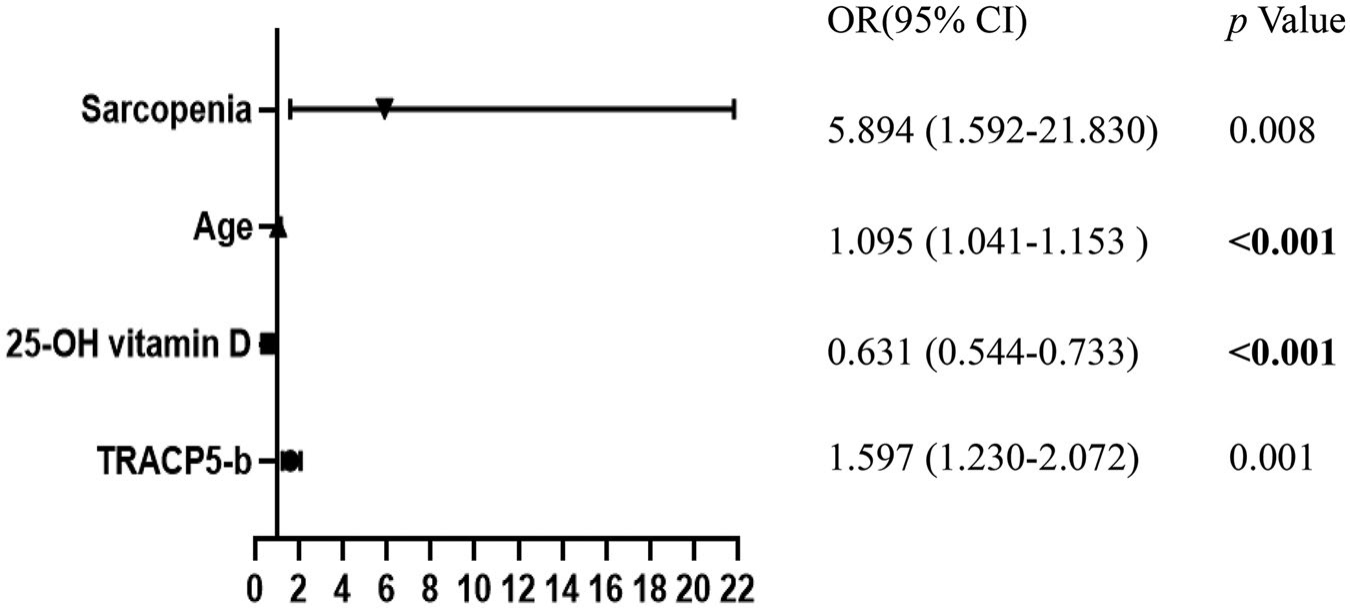
Ordinal logistic regression analysis was adjusted for sex, presence of diabetes mellitus, height, weight, BMI, albumin level and VDRA use. TRACP-5b, tartrate-resistant acid phosphatase 5b; 25-OH vitamin D, 25-hydroxyvitamin D; CI, confidence interval.

Figure 4 shows the factors associated with low BMD. In ordinal logistic regression analysis (modified by sex, diabetes, height, weight, albumin level and VDRA use), the odds ratio (OR) for low BMD was high for patients with sarcopenia (OR = 5.894, 95% CI 1.592–21.830, *p* < 0.01), older age (OR = 1.095, 95% CI 1.041–1.153, *p* < 0.001), higher TRACP-5b levels (OR = 1.597, 95% CI 1.230–2.072, *p* < 0.01), and lower 25-OH vitamin D levels (OR = 0.631, 95% CI 0.544–0.733, *p* < 0.001) (Table 6).

**Table 6.** Ordinal logistic regression analysis for BMD subgroups(normal BMD, Osteopenia, Osteoporosis).

	Estimate	SE	*p* Value	OR (95% CI)
ages	0.091	0.026	<0.001	1.095 (1.041–1.153)
TRACP-5b	0.468	0.133	0.001	1.597 (1.230–2.072)
BMI	0.018	0.082	0.83	1.018 (0.867–1.196)
Albumin	0.029	0.074	0.694	1.029 (0.890–1.190)
25-OH vitamin D	–0.46	0.076	<0.001	0.631 (0.544–0.733)
sex(male vs female)	0.015	0.484	0.666	1.015 (0.393–2.621)
presence of DM	0.605	0.652	0.354	1.831 (0.510–6.573)
sarcopenia	1.774	0.668	0.008	5.894 (1.592-21.830)
Alfacalcidol/Calcitriol use	−0.736	0.553	0.183	0.479 (0.162-1.416)
Paricalcitol use	−1.034	0.916	0.259	0.356 (0.059-2.141)

BMI: body mass index; TRACP-5b: tartrate-resistant acid phosphatase 5b; 25-OH vitamin D:25-hydroxyvitamin D; DM: diabetes mellitus; CI: confidence interval; SE: standard error.

Multivariate analysis was adjusted for sex, presence of diabetes mellitus, height, weight, BMI, albumin level and VDRA use.

## Discussion

Patients with CKD have a higher risk of fractures due to abnormal bone turnover, which includes high turnover (hyperparathyroid bone disease), low bone turnover or adynamic bone disease (ABD), osteomalacia and mixed uremic osteodystrophy, and is associated with high morbidity, mortality [19] and economic burden. This is the first study to evaluate the relationship between CKD-MBD and sarcopenia in patients on maintenance dialysis in China. An observational study demonstrated that patients with CKD-MBD may have a spectrum of bone disorders [1], and the loss of ALM leads to an increase in bone loss risk. As a result, dialysis patients also demonstrate bone loss and sarcopenia, which combination is termed osteosarcopenia [10]. In our study, HD patients had a higher proportion of Osteopenia and Osteoporosis than healthy controls, whether in the elderly or in the young age group. Patients on hemodialysis are at increased risk of BMD loss than the healthy controls. Among 130 HD patients, 36 were diagnosed with sarcopenia (27.7%), 44 were diagnosed with osteopenia (33.8%), 19 were diagnosed with osteoporosis (14.6%), and 23 were diagnosed with osteosarcopenia (17.7%). HD Patients are more likely to develop BMD loss. The prevalence of osteosarcopenia was 10.4% in males and 15.1% in females in a study of community-dwelling Chinese elderly individuals [20], and frailty, mortality, and fragility fractures were more prevalent among patients with osteosarcopenia [20–22]. Thus, in patients on hemodialysis, low muscle mass and bone loss should be monitored and treated to reduce adverse outcomes.

We observed that patients in the osteopenia and osteoporosis groups had lower muscle mass parameters (SLM, SMM, ASM and ASMI values) than those in the normal BMD group because skeletal muscle supports the bones. Ito et al. find that SMI was the independent factor that affected lumbar spine and femoral neck BMD in 50 HD patients [23]. Other studies reported positive correlations of ASMI with total BMD in patients undergoing HD [23–25]. Similarly, our results showed that a high prevalence of low BMD was associated with low muscle mass. Patients with sarcopenia levels of ASM had lower BMD. SMI was positively correlated with both lumbar spine BMD and femoral neck BMD in our study. This might be explained by the muscle–bone crosstalk. In human anatomy, bone and muscle are adjacent and thus share many common factors, including mechanical factors, chemical factors, genetic factors, and endocrine factors. The interactions between muscle and bone have been increasingly studied as a new research field. Myogenic and osteogenic cells originate from the same mesenchymal precursor, and genetic polymorphisms of several genes may influence osteoporosis and sarcopenia, such as androgen receptors, estrogen receptors, IGF-I, and vitamin D receptors [26,27]. Muscle tissues secrete myokines that affect bone metabolism, such as myostatin, IGF-1, IL-6, IL-15, FGF2 and osteoglycin. Meanwhile, osteocyte secrete osteokines prostaglandin E2,Wnt3a and sclerostin, osteoblasts secrete osteocalcin. All of these osteokines expressed by bone tissues may affect muscle metabolism [28]. Vitamin D [29], the growth hormone/insulin-like growth factor axis, and testosterone [30,31] are physiologically and pathologically important endocrine factors. Therefore, maintaining BMD depends indirectly or directly on muscle health.

In general, and among older adults, muscle health and BMD are positively correlated [32]. This is consistent with our research. We observed that HD patients with osteopenia and osteoporosis were more likely to be older than those with normal BMD, and older age was an independent risk factor for lower BMD in HD patients. In contrast, sarcopenia and bone loss in dialysis patients are caused not only by aging but also by their different lifestyles compared with the general population, which include exercise and protein intake decline, vitamin D deficiency, growth hormone resistance, decreased protein synthesis and accelerated protein catabolism during a dialysis session. Previous studies demonstrated that osteoporosis and sarcopenia in geriatric inpatients are linked to nutritional deficits. Even though we found that patients in the osteopenia and osteoporosis groups had lower albumin levels than patients in the normal BMD group, lower albumin was not an independent factor of BMD. This may be due to dialysis patients in our centre receiving better nutritional support. In this study, the albumin level in most dialysis patients was not very low.

No differences in laboratory parameters were found among the groups with normal BMD, osteopenia, and osteoporosis, including those related to Hb, hsCRP, and bone mineral metabolism (calcium, phosphorus, PTH and bAP). We used various diagnostic and therapeutic strategies to appropriately prevent and manage the CKD-MBD status of patients undergoing HD, therefore the CKD-MBD-related data were well controlled. This might explain why these markers were not independent risk factors associated with BMD in the present study. Kazama JJ found that PTH level was negatively correlated with cortical bone thickness, but this relationship disappeared among those with intact PTH levels <1000 pg/mL [33]. In our cohort, patients with PTH levels >1000 pg/mL were rare. Bone turnover markers (BTMs) may also inform BMD status and predict BMD change. BTMs have been independently associated with BMD in CKD patients [25]. Moldovan et al. [34] observed that high OPG levels correlate with higher bone mineral density since osteoprotegerin inhibits osteoclast activity. TRACP-5b is the only bone resorption marker that is not cleared by the kidneys, making it ideal for assessing bone resorption in chronic HD patients. Additionally, TRACP-5b, which is released into the circulation, is a specific biomarker of osteoclast number and activity [35]. Our study showed that BMD was associated with bone resorption markers (TRACP-5b), and a higher TRACP-5b level was an independent risk factor for lower BMD. Denosumab, which inhibits the formation and activity of osteoclasts, and decreases bone resorption, was demonstrated safety and efficacy in treating osteoporosis in hemodialysis patients, and TRACP-5b was used for follow-up efficacy [36].

As both muscle and bone express vitamin D receptors, vitamin D plays a crucial role in muscle and bone development [37]. Aside from its effect on calcium absorption, vitamin D is associated with myoblast differentiation and damage to type II myofibres [38]. As CKD progresses, several biochemical changes occur, including hyperphosphatemia, hypocalcemia, increased fibroblast growth factor 23 (FGF-23) and PTH levels, as well as vitamin D concentrations [39]. CKD has been associated with increased susceptibility to vitamin D insufficiency and deficiency states. We observed that the serum 25(OH)D level was significantly different between the three subgroups. Deficiency or insufficiency of vitamin D was prevalent in all subgroups. Lower 25(OH)D was an independent risk factor for lower BMD. Today experts use the terms “kidney - bone - vascular axis” in CKD, and some studies find that renal osteodystrophy (ROD) was predictive of Vascular calcifications (VCs) development [19]. Vitamin D supplementation regimens should be used carefully to reduce the fracture risk in patients undergoing hemodialysis, meanwhile without an increase in the burden of vascular calcifications.

Therefore, it is important that the assay be used for HD patients to monitor BTMs, especially TRACP-5b and vitamin D levels, which might help identify patients who might benefit the most from bone-preserving measures. Furthermore, appropriate medications can be used to achieve optimal 25OH-D and TRACP-5b levels and to control the incidence of sarcopenia and OP while on HD.

However, there are limitations to this study. First, the sample size was relatively small. Second, this was a cross-sectional study, so causality cannot be inferred. A prospective, longitudinal study is needed to investigate the relationship between bone loss and its influencing factors in CKD-MBD. Larger multicenter cohort studies and corresponding basic research are needed to elucidate the pathogenesis and influencing factors of BMD loss, with long-term follow-up to explore its impact on fracture risk and mortality risk.

## Conclusions

The prevalence of SP, OP and osteosarcopenia was high in this single-center cross-sectional study of patients on maintenance dialysis in China. Individuals with advanced age, sarcopenia, lower 25-OH vitamin D levels and higher TRACP-5b levels tended to have a higher risk of BMD loss, suggesting that HD patients with bone loss should be assessed for concomitant SP and BTM. Adequate intake of vitamin D and control of TRACP-5b levels will help reduce the occurrence and progression of osteopenia/sarcopenia in HD patients. Therefore, it may be time to reevaluate the diagnostic and therapeutic protocols for bone loss prevention in CKD-MBD. Novel therapeutic approaches to treat musculoskeletal pathologies should be explored and novel approaches with integrated management need to supplement the traditional management of CKD-MBD.
